# Patient knowledge, attitudes and practices on chronic wound infections in Tanga Regional Referral Hospital, Tanzania; A qualitative study

**DOI:** 10.1371/journal.pgph.0004698

**Published:** 2026-02-24

**Authors:** Aleena Dawer, Victor Msengi, Pendo Magili, John P. A. Lusingu

**Affiliations:** 1 National Institute of Medical Research Tanga Centre, Hospital Rd, Tanga, Tanzania; 2 Georgetown University, Washington D.C., United States of America; 3 Tanga Regional Referral Hospital, Hospital Rd, Tanga, Tanzania; University of Oxford, UNITED KINGDOM OF GREAT BRITAIN AND NORTHERN IRELAND

## Abstract

Bacterial wound infections contribute significantly to global mortality, with nearly half of contaminated wounds progressing to chronic states, which can result in amputations. In Tanzania, the situation is worsened by the misuse of antibiotics and the rise of drug-resistant bacteria, alongside patients’ varied understanding of wound care management. This qualitative study explores patient knowledge, attitudes, and practices (KAP) regarding chronic wound infections in Tanzania. A sample of 15 patients was selected from both genders, representing a wide range of ages and wound severities. Semi-structured interviews were conducted in Swahili at the Tanga Regional Referral Hospital (TRRH) from October 2023 to April 2024. Interviews used open-ended questions on dietary habits, traditional medicine, medication adherence, and wound care practices. Social demographic data were collected to contextualize patient experiences. Interviews were audio-recorded, transcribed verbatim, and analyzed via Nvivo. Thematic analysis of the interviews identified six major themes: delays in treatment-seeking, inadequate wound management, reliance on ritual practices, poor eating behaviors, challenges in coping with chronic wounds, and inadequate healthcare services. Delays stemmed from misjudging wound severity and reliance on traditional healers. Financial constraints contributed to inadequate wound management, with many patients turning to traditional remedies. Nutritious food access was limited, primarily due to financial and availability constraints. Patients faced substantial physical, emotional, and financial burdens. Long wait times and inconsistent care were also major barriers to effective wound management. Chronic wound infections are exacerbated by systemic healthcare shortcomings and limited patient education. Insights from patient perspectives emphasizes the need for proper wound management, understanding of antimicrobial resistance (AMR), and addressing cultural beliefs and resource constraints. Patient-centered models can foster more effective and sustainable models of wound care in the community setting.

## Introduction

Chronic wound infections are a pervasive global health issue, significantly reducing patient quality of life and placing a substantial burden on healthcare systems [1]. Defined as wounds that fail to heal within three months, chronic wounds often persist due to a complex interplay of biological, socioeconomic, and healthcare-related factors. These infections are a leading cause of major lower-limb amputations, with systematic review studies showing that up to 50% of patients die within five years of undergoing such procedures [2]. Diabetic foot ulcers (DFUs) alone account for approximately 85% of these amputations worldwide [3]. Yet many of these outcomes are preventable with timely diagnosis and effective treatment.

However, the patient perspective is essential to effectively address the challenges of chronic wound management. Current research highlights a significant deficiency in patient understanding about proper wound care and the appropriate use of antibiotics, leading to suboptimal treatment outcomes and increased risk of AMR. For instance, 20% of Europeans reported using antibiotics to self-treat influenza, despite antibiotics being ineffective against viral infections [4]. Similarly, in Kenya, a detailed analysis in selected hospitals in Kajiado County found correlations between septic chronic wounds with individual understanding, compliance with prescribed drug dosages, alcohol consumption, and smoking habits [5]. In the U.S., a study found that 38% of patients were unaware of proper wound dressing techniques and 58.7% lacked education on wound cleansing after hospital discharge [6]. These patterns point to a widespread gap in patient-centered care and education.

In Tanzania, these challenges are further magnified by resource limitations and high rates of antibiotic misuse. In Tanga, a coastal region in northeastern Tanzania, antimicrobial resistance (AMR) complicates wound management even further and is provided in the quantitative analysis of this study [7]. Tanga, in particular, faces high rates of poverty, with districts like Mkinga reporting that over 93% of residents live below the poverty line [8]. A regional survey of health laboratories found widespread limitations in diagnostic capacity, including a lack of trained personnel and basic equipment with only 3% of facilities being able to perform culture and sensitivity testing, which is critical for guiding wound infection treatment [9]. These systemic gaps hinder effective care for both acute and chronic conditions. While DFUs are a major contributor to global chronic wound burden, the patient population in this study presented with a range of chronic wound etiologies, including diabetes, fungal infections, and trauma-related wounds. Despite the clinical importance of managing chronic wounds, there is a lack of published research exploring how Tanzanian patients perceive and manage wound infections during hospitalization. This study addresses that gap by conducting in-depth, semi-structured interviews with hospitalized patients in Tanga Regional Referral Hospital, contributing qualitative insight into how patients perceive and manage chronic wound infections.

## Methods

### Ethics statement

The proposed study is grounded in the ethical principles of beneficence, non-maleficence, and respect for autonomy. The anticipated benefits of the study, which include enhancing knowledge about chronic wound infections and potentially developing more effective treatment strategies, justify the ethical conduct of the study. The study protocol was endorsed through rigorous scientific ethical review by the National Institute of Medical Research (NIMR) (ethical approval NIMR/HQ/R.8a/Vol. IX/444 granted on 27/10/2023). All participants signed a written informed consent prior to inclusion. All methods were performed in accordance with the relevant guidelines and regulations.

### Study aim, design and selection criteria

This study employed a phenomenological qualitative approach conducted from 27/10/2023- 01/04/2024 to explore the experiences, challenges, and perceptions of adult patients suffering from chronic wound infections at the Tanga Regional Referral Hospital (TRRH). The selection criteria focused on all admitted patients of ages older than 18 and sexes exhibiting chronic wound infections. These criteria were chosen to provide comprehensive insights into the factors affecting chronic wound management in this clinical setting.

### Sample size and population

The focal point of this study was the diverse group of patients admitted to TRRH with chronic wounds. This population includes both males and females who have been afflicted with chronic wounds. 15 patients were selected for in-depth interviews (IDIs), ensuring a mix of age, gender, and wound severity to capture diverse experiences. Although TRRH admits an average of 28 patients per month for chronic wounds, many were excluded due to being under 18, having cognitive impairments, being medically unstable, or being discharged before interviews could be conducted. No eligible patient declined participation. The chosen number of patients was believed to be sufficient to reach thematic saturation based on consultation with a qualitative expert. As interviews progressed, no new themes emerged, indicating that key patterns were captured and saturation was reached.

### Study setting

TRRH, commonly referred to as Bombo Regional Hospital, is a prominent hospital located in Tanga, Tanzania. Established during the German colonial period in the 1890s to combat high malaria mortality rates, TRRH has since evolved into a key medical facility. The hospital, which admits an average of 28 patients per month for chronic wound infections, serves as a crucial center for both routine and specialized healthcare services in the region.

### Sampling strategy

A consecutive sampling strategy was adopted for this study. Between 27/10/2023- 01/04/2024, all patients meeting the inclusion criteria were approached for participation until the desired sample size was achieved. Patients were screened daily by the research team using ward logs and direct referrals from clinical staff. Eligibility criteria included: (1) age ≥ 18 years, (2) diagnosis of a chronic wound persisting for more than three months, (3) ability to provide informed consent, and (4) cognitive stability to participate in an interview.

While all eligible patients were approached, maximum variation purposive sampling was used within the consecutive framework to ensure heterogeneity across key characteristics: age, gender, wound type, wound duration, and underlying medical conditions. The goal was to capture a wide range of lived experiences and treatment trajectories.

### Data collection

Participants had variability in age, gender, wound severity, and treatment delays which was considered related to the narratives shared. While no statistical comparisons were conducted, participant demographics were entered as attributes in NVivo and used in matrix coding queries to explore whether patterns emerged across subgroups. This allowed us to examine potential variation while remaining consistent with a qualitative interpretive approach. Individual interviews were chosen over focus groups to encourage candid and uninfluenced accounts of patient experiences with chronic wounds and healthcare interactions.

Participants were thoroughly informed about the study’s objectives and procedures, and informed consent was obtained from each participant. The interview guide was designed to cover key domains within 10–15 minutes to respect patients’ clinical status and minimize respondent burden. However, interviews were conducted in a flexible, participant-led manner, with open-ended questions and follow-up probes used to encourage elaboration. Most participants extended the conversation, and final recordings typically ranged from 25 to 35 minutes in duration. The interviews were conducted in Swahili to reduce language and cultural barriers and facilitate more natural dialogue. The interview guide was developed using constructs from Kleinman’s Explanatory Models of Illness and the Health Belief Model, specifically perceived severity and perceived barriers, to explore how patients understand their wounds and treatment choices. Principles of constructivist grounded theory guided data collection and analysis, allowing for emergent, participant-led insights. The interviews followed a structured guide divided into two sections. The first section explored dietary habits, use of traditional medicine, medication adherence, and wound care practices. The second section focused on challenges faced by patients, their suggestions for improvement, and their perspectives on their condition and treatment. The guide was pilot tested on two patients and refined to allow more open-ended, participant-led responses. Our interviewee used follow-up probes and adapted questions as needed. Reflexive field notes were documented in NVivo, noting contextual cues, participant dynamics, and researcher influence, and were reviewed during the analysis.

Sociodemographic data, including age, gender, residence, education level, occupation, religion, and wound type and duration, were collected to provide a comprehensive understanding of patient experiences. The study explored both practical treatment aspects and personal, subjective perspectives.

A significant emphasis was placed on the comfort and privacy of participants during interviews. Participants were allowed to choose their preferred location for the interview, ensuring that their perspectives and experiences were shared freely and without interference. Data recording involved the use of audio recorders and note-taking. Post-interview, the audio-recordings were transcribed verbatim and translated from Swahili into English. Two bilingual co-authors (V.M, P.M) independently cross-checked each transcript against the original audio to ensure accuracy and fidelity to participants’ responses. Careful attention was paid to idiomatic expressions and culturally relevant terminology throughout the process to preserve participant meaning.

### Data analysis

Thematic analysis was conducted using NVivo v.14 software, following an iterative process. An initial codebook, outlining deductive codes, was developed based on the study’s predefined objectives. Two researchers independently coded a subset of transcripts to refine the codebook, resolve discrepancies, and ensure reliability.

Four main deductive themes were confirmed during coding, while inductive analysis revealed two additional emergent themes. Constant comparison across transcripts ensured thematic saturation. NVivo’s coding queries and matrix searches systematically grouped excerpts and explored relationships between themes and participant characteristics.

Coded data were synthesized into overarching categories and sub-themes, supported by illustrative quotations. Memo-writing was used throughout the process to document analytic decisions and track evolving interpretations. This rigorous, multi-step approach ensured that the final thematic structure provided a credible representation of participants’ experiences with chronic wound management.

## Results

Fifteen patients participated (age range: 35–80 years; median: 55); with a balanced gender distribution (8 males, 7 females) and the majority (93%) presenting leg wounds. The etiologies varied: some had wounds arising from DFUs, while others stemmed from traumatic injuries, fungal infections, or unknown causes that worsened over time. Wounds were reported to have persisted before hospitalization, ranging from three months to over three years. Delays in seeking formal care were frequently linked to prior reliance on traditional remedies, lack of perceived urgency, or financial constraints. Participants described trying multiple treatments, such as herbal leaves, over-the-counter creams, or advice from peers, before eventually presenting at TRRH. These prolonged pre-hospital management attempts may have contributed to poor wound healing and increased the risk of infection or complications. Most participants had only primary school education (73.3%), and nearly half (46.7%) worked in agriculture, aligning with regional workforce patterns (47.5% of women and 51.9% of men in Tanga work in farming) [10].

The sample’s older median age (55 vs. Tanga’s ~25 years) reflects chronic wounds’ association with aging [11]. Religious and educational distributions also reflected regional norms [10].

This quantitative Table 1 presents the social demographic characteristics of the patients interviewed at TRRH. Included are data on age, gender, religion, occupation, and education level. While these characteristics were descriptively analyzed, thematic patterns in interviews were generally consistent across these demographic groups. One exception was a healthcare worker who demonstrated biomedical knowledge. However, most themes were shared across the sample, underscoring the potential systemic nature of wound care challenges.

**Table 1:** Demographic characteristics of TRRH patients interviewed.

Characteristic, n (%)	Interview, N = 15
Patient age, years (median)	35-80 (55)
Gender female	7 (46.7)
Religion, n (%)	
Christian	8 (53.3)
Muslim	7 (46.7)
Occupation, n (%)	
Farmer	7 (46.7)
Retired	4 (26.7)
Carpenter	1 (6.7)
Healthcare Worker	1 (6.7)
Housewife	1 (6.7)
Teacher	1 (6.7)
Education level of interviewees, n (%)	
None	1 (6.7)
Primary	11 (73.3)
Secondary	2 (13.3)
College	1 (6.7)

Table 2 outlines major themes and their related codes from patient interviews, covering areas such as delays in seeking treatment, wound management, ritual practices, eating behaviors, coping with chronic wounds, and challenges with healthcare services. These themes capture various factors affecting patient care and experience at TRRH. The analysis initially identified four themes, with two additional inductive themes—“Living and Coping with Chronic Wounds” and “Inadequate Healthcare Services”. As the interviews progressed, no significant new themes emerged. Participants’ responses consistently reiterated previously identified ideas, indicating that key patterns in the data had been captured and that data saturation had been reached.

**Table 2:** Summary of themes and codes.

Themes	Codes
Delay in treatment-seeking	• Limited knowledge of wound symptoms requiring immediate medical attention
Wound management	• Lack of priority for wound management • Poor understanding of wound care guidelines
Ritual practices	• Seeking care from traditional healers • Belief in the efficacy of traditional treatments
Eating behavior	• Loss of appetite • Limited knowledge of balanced nutrition for wound healing
Living and coping with chronic wounds	• Anxiety • Stress • Financial hardship • Discomfort in daily life
Inadequate healthcare services	• Long waiting times to see specialists • Use of unsuitable or poorly equipped treatment centers • Insufficient or inconsistent training among healthcare providers for wound care

### Delay in treatment-seeking

Postponed hospital treatment was a significant theme among patients with wound infections. Most patients expressed a lack of understanding of severe wound symptoms, leading them to attempt self-care or seek help from traditional healers. Hospital care was often only sought once the wound had significantly worsened. The findings here indicate an urgent need for enhancing public awareness about recognizing wound symptoms early and understanding the risks of delayed medical care, including the potential for severe infections and the spread of antibiotic-resistant strains.

*“It took like six months without going to the hospital…” (P2, Male, 49 years old)*

*“Unfortunately, I thought it was a fungus [and] treated it with antifungal drugs. Only when my leg became swollen and turned brown, then I knew there was a problem…” (P9, Male, 50 years old)*

*“I stayed at home for a while and went to the pharmacy after seeing that my leg was swollen. I got five injections for five days without success, so I went to the hospital. After arriving they said, “the leg is already in a bad condition” (P13, Male, 58 years old)*

### Wound management

Participants described their methods of wound care both before and after hospital visits. Their responses revealed limited familiarity with formal wound care guidelines. Many reported using readily available materials, such as non-sterile cotton swabs or pieces of cloth, to clean and cover their wounds. For example, P1 described cleaning and covering the wound with a cloth. While participants often used whatever materials were available, using non-sterile coverings raises concerns about potential infection risks.

Financial constraints also played a critical role in hindering adherence to recommended wound care practices. Participants recounted how financial constraints forced them to skip necessary dressing changes at the clinic, highlighting the harsh reality of having to choose between proper medical care and other daily expenses. Such decisions not only compromise wound healing but also escalate the risk of complications. One patient illustrated the dire consequences of inadequate wound care in the context of chronic conditions like diabetes, which can lead to severe outcomes such as amputations.

*“Ah, for the first month I cleaned it every day, but in the following month, I would skip a day and then go dress it… I was unable to go frequently because of the financial challenge… for dressing and transport cost to reach the health center every day…” (P4, Female, 35 years old)*

### Ritual practices

Patients frequently turned to traditional healers and plant-based remedies for managing chronic wounds, often attributing their conditions to supernatural causes like witchcraft. This theme is critical as it reveals the cultural belief systems influencing health-seeking behaviors for chronic wounds.

Participants often preferred traditional healers over medical facilities. One male patient described visiting eight healers over three years in search of effective remedies. Despite his efforts, the treatments worsened his condition, highlighting the desperation and hope that drove his reliance on these practices. The diverse explanations for his wound given by different healers, ranging from mystical interpretations like “two birds with two beaks” causing the wound or having “offended mermaid children,” reflect the varied and often complex belief systems surrounding illness in Tanzania.

The use of plant-based remedies further illustrates the reliance on traditional healing practices. For instance, one diabetic patient used *Mzambarao* bark (*Java Plum* or *Syzygium cumini*), recommended by another patient, believing it would reduce symptoms like frequent urination. Similarly, another participant applied a leaf to his wound after dissatisfaction with hospital medicines, trusting in natural treatments perceived as safer and more effective than conventional care.

Participants’ reliance on traditional healers and traditional remedies not only demonstrates a cultural inclination but also possibly reflects underlying issues in the healthcare system, such as accessibility, affordability, and trust. The persistence of these practices, despite instances of adverse effects, underscores the profound influence of cultural and traditional beliefs in health management, especially in communities where such practices are deeply ingrained.

While some traditional practices delayed access to conventional medical treatment, certain plant-based remedies used by participants may hold genuine therapeutic value. For instance, Mzambarao bark has been studied for its potential antidiabetic and anti-inflammatory properties [11]. The use of traditional treatments reflects not just cultural preferences but also historical knowledge of local medicinal plants. Integrating evidence-based understanding of traditional remedies into healthcare could improve delivery by fostering trust and offering more culturally attuned care.

*“I visited like eight traditional healers, used traditional medicine for three years… the condition worsened … you apply medicine, the leg becomes sore and itchy…” (P2, Male, 49 years old)*

*“No, I don’t use any herbal medicines” (P4, Female, 35 years old)*

*“The second [healer] spoke of two birds with two beaks, saying that’s why I have sores here and there, that’s what’s troubling you. (Laughter)…the third claimed spirits wanted me to become a traditional healer…another said I stepped on mermaid children…at a crossroads…” (P2, Male, 49 years old).*

*“I used the bark of a tree called Mzambarao… effectiveness was…equivalent to the pills because it lowered the sugar level faster…” (P6, Male, 67 years old).*

*“Every time I take these hospital medicines I don’t get relief, I met a gentleman, he said let me give you medicine. He gave me a leaf and I chewed it and put it on my wound.” (P9, Male, 50 years old)*

### Eating behavior

Participants’ accounts revealed that economic hardship and illness-related appetite loss were major barriers to maintaining diets that support wound healing. Some participants acknowledged the importance of food in recovery but faced significant challenges in accessing adequate nutrition. Protein-rich foods such as meat were described as unaffordable, and several participants reported skipping meals due to financial constraints.

For instance, one participant mentioned eating meat rarely due to financial constraints, another reported regularly skipping lunch, and a third described relying on porridge and struggling to stabilize blood sugar despite eating minimally.

*“I have never followed any diet, I eat ugali, rice and sometimes miss meals completely…I eat meat but once in a while depending on the economic situation, if I get it today I might go a whole month without it again.” (P2, Male, 49 years old).*

*“At night... we eat light foods like bananas… or cassava with tea.” (P1, Female, 64 years old)*

*“I often don’t eat at lunch.” (P4, Female, 35 years old)*

*“In the morning, I can only drink porridge...And eat nothing until the next morning...I can’t taste food...But surprisingly, the sugar level doesn’t stabilize.” (P5, Male, 80 years old).*

### Inadequate healthcare services

Poor healthcare services can significantly hinder progress in health improvement, particularly when individuals with serious health conditions, such as chronic wounds, seek treatment and discover that immediate care is unavailable. Participants’ experiences reveal a range of issues from delayed treatment to inadequate guidance on wound care, painting a picture of a healthcare system struggling to meet the needs of patients with complex conditions.

Delays in treatment were a recurring issue. One patient described a critical delay upon hospital arrival that worsened his condition, while another recounted waiting four days without treatment at a district hospital before being transferred to a referral center.

Inadequacies in wound care and management advice were also evident. Several participants reported a lack of detailed instructions from healthcare workers (HCWs) on cleaning and managing their wounds. This lack of guidance often left patients to rely on their understanding or alternative remedies. One account described a wound worsening despite receiving basic care at a health center spotlights the complexity of wound management, where both healthcare guidance and the patient’s ability to follow at-home care practices play critical roles in outcomes.

Participants also reported inconsistencies in treatment protocols across facilities. P4, for instance, described differing approaches, from povidone and metronidazole to honey dressings. This lack of standardization and clear communication undermines patient trust and contributes to poor outcomes, as seen with one patient, who ultimately required a toe amputation due to inadequate initial care and follow-up.

*“Since I arrived here on Tuesday evening, they have already set their treatment time table only for Tuesday and Wednesday of next week. So it bothered me and it was difficult to get treatment and I see that the bacteria are attacking more and more now…” (P9, Male, 50 years old)*

*“I went to the district hospital, I didn’t get any treatment there. I stayed there for four*

*days, so I had to come back to the referral hospital …” (P10, Male, 55 years old)*

### Living and coping with chronic wounds

The testimonies reflect a spectrum of challenges, from physical and emotional struggles to socioeconomic constraints, all of which paint a vivid picture of the multifaceted burden of a chronic wound. Patients frequently expressed profound anxiety, particularly about potential amputations. One participant described the distress of a foul-smelling discharge affecting his social interactions and self-esteem.

Physical challenges were also prominent, for example, a female patient shared how swollen feet make it difficult to walk or wear shoes. This physical limitation represents just one aspect of the broader impact on daily life and mobility. Another recounted a harrowing experience of unconsciousness and severe burns, revealing the drastic, life-altering consequences of chronic conditions and accidents, which are compounded when layered with chronic wound management.

Economic constraints were another critical aspect, with several discussing the financial burdens of treatment and the reliance on their children to support them. This economic stress can lead to delayed or inadequate treatment, exacerbating wound progression. One patient detailed the challenge of adhering to treatment schedules and the demoralization felt when treatments do not yield immediate results, reflecting the psychological toll of managing a chronic condition.

A chronic wound is not just the physical pain but also the psychological distress, social challenges, and economic burdens faced by patients. All patients (n = 15) expressed the emotional toll of anxiety and isolation that ripples into their lives, affecting their relationships.

*“Ah, challenges, truly...just worries. Anxiety because often with diabetic wounds, you hear about people having their legs amputated…you just feel the pain, it’s like pricking - Ah - it’s sharp. Secondly, the fluid it releases has a bad smell, so when you sit with others... you find flies following you, you can’t sit comfortably.” (P6, Male, 67 years old)*

*“My body becomes very heavy, I can’t handle it…For example, I might go outside, but it’s a challenge. I might fall…It feels like my legs are weakening, yes. And, you might use the toilet but then find it hard to get up. In short, I can’t manage on my own.” (P5, Male, 80 years old).*

*“Ah, I don’t really understand the medications I take.” (P10, Male, 55 years old).*

## Discussion

This study fills a critical gap in understanding patients’ knowledge, attitudes, and practices (KAP) regarding chronic wound infections in Tanzania, an area with limited prior research. While studies in Sub-Saharan Africa (SSA) have examined HCW perspectives on wound care, such as in Ghana [12], few have focused on patients’ lived experiences, which is essential for improving wound management practices. Building on global findings, this study highlights the unique challenges shaped by the sociocultural and healthcare dynamics of a specific region within SSA.

Globally, patient KAP regarding wound infections share commonalities but also reveal regional differences. For example, a Canadian study [13] reported the significant emotional and psychological toll of chronic wounds, with patients expressing frustration, anxiety, and concerns about delayed healing. Similarly, Tanzanian participants in this study described fear of amputation and the stigma associated with chronic wounds, including embarrassment over foul odors and flies, which impacted their social interactions and mental well-being. Both studies also emphasized the challenge of self-managing symptoms, with pain, fatigue, and difficulty concentrating being debilitating and affecting daily activities. However, a notable difference was that the Canadian study revealed numerous concerns among patients regarding burdening their family members, a sentiment rarely expressed in the Tanzanian context.

### Treatment disparities

In addition to psychosocial experiences, disparities in available treatment options further shaped patient outcomes and practices in chronic wound management. While parallels exist in the emotional and psychological burdens experienced by patients globally, stark differences in treatment availability highlight inequalities in healthcare infrastructure. Canadian patients had access to advanced interventions such as sharp debridement and compression bandages, whereas Tanzanian participants relied on basic treatments like honey dressings and, in severe cases, amputations. The use of honey as a wound dressing agent at TRRH mirrors findings by previous research [14] which documented its widespread use in SSA for its natural healing properties. This integration of traditional and allopathic practices underscores the need for context-specific solutions in resource-limited settings.

### Cultural and economic influences on traditional medicine

A predominant theme in this study was the reliance on traditional healing practices, driven by cultural beliefs and economic constraints. Similar to findings in previous studies [15,16], patients turned to plant-based remedies due to their perceived effectiveness, affordability, and alignment with cultural norms. However, the lack of standardization in traditional practices, such as inconsistent dosage measurements, raises concerns about safety and efficacy. In a meta-literature review [17], it was reported that 80% of Africa’s population relies on medicinal plants as their primary form of medication, a finding reflected in this study, where many patients sought traditional remedies before transitioning to hospital treatments.

Economic factors further shaped healthcare decisions, with limited finances driving reliance on affordable traditional medicine over formal medical care. This aligns with previous observations [18] that inaccessible healthcare facilities and financial constraints are critical barriers to seeking professional treatment.

Beyond cost, participants’ references to supernatural causes, such as mermaid children or spiritual interference, may reflect broader mistrust in biomedical explanations of illness. As outlined by Kleinman’s Explanatory Models of Illness, patients interpret disease through sociocultural lenses that may differ significantly from biomedical paradigms. Patient accounts revealed how treatment choices and trust in healthcare were shaped by beliefs in spiritual causality as well as peer-shared remedies passed down through community advice and lived experience.

Addressing these belief systems requires more than top-down medical instruction to patients: it demands culturally sensitive engagement. Recognizing traditional medicine’s historical role while guiding patients toward safe, evidence-based care can build trust. For instance, community outreach could validate culturally familiar remedies like Mzambarao bark, while offering biomedical context.

Rather than viewing traditional and biomedical systems as oppositional, integration may offer a path forward. Cross-training initiatives, where traditional healers are trained to identify red flag symptoms and refer patients early, and biomedical providers learn about culturally meaningful remedies, can reduce care fragmentation and foster mutual respect. Such models may ultimately enhance continuity of care and improve outcomes in resource-limited settings.

While the specific remedies varied by context, reliance on traditional healing practices is not unique to Tanzania. For example, a study in Saudi Arabia [19] found that cultural practices for wound healing included the use of incense (36.8%), coffee beans (24.3% believing they could stop bleeding), and Sabkha (19% using it as an anti-inflammatory remedy). Such findings suggest that while traditional medicine is widespread across different regions, the specific substances, practices, and cultural interpretations of healing vary.

Despite standardization challenges, emerging evidence highlights the pharmacological potential of many traditional remedies. Ethnobotanical research in sub-Saharan Africa has documented over 2,000 medicinal plants with therapeutic properties, including antimicrobial, anti-inflammatory, and wound-healing effects [20]. A Sri Lankan study similarly reported that 68.1% of patients initially used “hand medicines” for diabetic wounds [21]. With further study and careful integration, these remedies could complement formal healthcare systems, enhancing culturally appropriate care and expanding treatment access in resource-limited settings.

### Dietary practices and nutrition

This study emphasized the major role that financial constraints and food accessibility challenges played in shaping participants’ diets during wound recovery. While many recognized the importance of nutrition for healing, they struggled to afford protein-rich or nutrient-dense foods. Limited financial resources often resulted in inadequate dietary intake, with patients relying on inexpensive staples like porridge, cassava, and bananas and frequently skipping meals altogether. This observation parallels findings from a Colombian HIV study [22], where 61.8% of participants faced similar challenges in food selection and purchasing due to financial and informational barriers. For improved recovery outcomes, interventions must combine patient education on nutrition’s role in wound healing with systemic efforts to increase affordability and access to high-protein foods.

### Barriers in healthcare access

Another critical challenge was delayed access to healthcare, a global barrier in managing chronic wounds. Prolonged wait times for treatment, criticized in multidisciplinary wound clinics in Canada [12], were also present in Tanzania, where participants reported significant delays in receiving care. In Tanzania, delays were often linked to long travel distances to hospitals, limited availability of specialized wound care services, and financial constraints that delayed initial care-seeking. In some cases, inefficiencies in the referral system further compounded these delays, with patients seeking care at multiple facilities before receiving appropriate treatment.

The consequences of delayed care were severe; participants in this study described irreversible damage, including infections worsening to the point of requiring amputations. Similar patterns were observed in a study from Sri Lanka, where 21.7% of patients undergoing delayed care ultimately required amputation [21]. These compounding delays worsen patient outcomes, highlighting the urgent need for structural reforms to ensure timely and equitable wound care access.

### Gaps in patient education

A recurring theme across studies was the limited engagement of HCWs in patient education. One transnational review of leg ulcer treatment adherence [23] reported that only 50% of nurses provided health education to leg ulcer patients. This trend is echoed in this study, where participants described insufficient guidance on wound care and lifestyle management. Similarly, misconceptions about wound care were observed in other contexts; for example, in the Saudi study [20], 41.5% of participants mistakenly believed that showering delayed wound healing, highlighting the importance of medical education and engagement with HCWs.

Such misconceptions, compounded by limited professional guidance, highlight the importance of investing in HCW training and allocating resources to empower patients through education. Globally, research on patient KAP in the context of wound infections has largely been conducted outside of East Africa, limiting its relevance to the region’s unique challenges. By capturing patient perspectives from Tanzania, this study offers valuable patient insights grounded in the regional context; highlighting the need for context-specific interventions and laying a foundation for future research in similar resource-limited settings.

### Limitations

While this study provides valuable insights into wound care challenges in resource-limited settings, several limitations should be noted. This study was conducted with a small sample of patients from a single facility, limiting the findings’ generalizability. We do not claim to represent the full spectrum of wound types, severities, or patient experiences across Tanzania. Instead, the study serves as an exploratory blueprint for future research by offering initial insights into the healthcare-seeking behaviors, coping strategies, and wound management challenges faced by patients in resource-limited settings.

The qualitative approach, while ideal for capturing patient perspectives in depth, requires validation through larger, mixed-methods studies. Limitations inherent to qualitative research include potential social desirability bias during interviews, researcher influence during coding and interpretation, and risks for translation accuracy from Swahili to English. These were mitigated through pilot interview testing, reflexive memo-writing, and bilingual collaborative transcription and cross checking to ensure cultural nuance and data fidelity. Additionally, we were unable to verify the names of all health facilities visited by participants prior to TRRH, as many sought care in informal or unnamed village clinics.

These limitations notwithstanding, the study offers foundational knowledge about healthcare-seeking behaviors and coping strategies in Tanzania’s chronic wound population, a previously understudied area.

## Conclusions

This study reveals critical gaps in Tanzania’s chronic wound care landscape, particularly regarding health literacy, healthcare access, and the complex interplay of psychological, cultural, and socioeconomic barriers. Through patient-centered exploration, it identifies actionable themes to guide future interventions for improved outcomes.

While offering an early in-depth exploration of these challenges in the Tanzanian context, our findings underscore the need for three key developments: expanded research to validate these patterns across diverse clinical settings, culturally-adapted wound care protocols that bridge traditional and biomedical approaches, and health system reforms addressing both affordability and care coordination. These exploratory results establish an essential foundation for developing context-specific solutions that address the complex realities of chronic wound management in resource-limited environments, where patient needs intersect with structural healthcare constraints in particularly consequential ways.

## Supporting information

S1 Text Appendix A: Voices of Experience: Thematic Categorization of Participant Testimonies on Health Challenges and Practices. (DOCX)

S2 Text Semi-Structured Survey on Wound Healing Practices. (DOCX)

S1 Checklist COREQ (COnsolidated criteria for REporting Qualitative research) Checklist. (DOCX)

## References

[1] Olsson M, Järbrink K, Divakar U, Bajpai R, Upton Z, Schmidtchen A, et al. The humanistic and economic burden of chronic wounds: A systematic review. Wound Repair Regen. 2019;27(1):114–25. doi: 10.1111/wrr.12683 30362646

[2] Thorud JC, Plemmons B, Buckley CJ, Shibuya N, Jupiter DC. Mortality after nontraumatic major amputation among patients with diabetes and peripheral vascular disease: a systematic review. J Foot Ankle Surg. 2016;55(3):591–9. doi: 10.1053/j.jfas.2016.01.012 26898398

[3] World Diabetes Foundation. Diabetes foot care - Step-by-Step. Bagsværd, Denmark: World Diabetes Foundation; Available from: https://worlddiabetesfoundation.org/what-we-do/projects/wdf03-0056/

[4] European Commission. Special Eurobarometer 407: Antimicrobial resistance [Internet]. Brussels: European Commission; 2013. Available from: https://health.ec.europa.eu/system/files/2016-11/ebs_407_sum_en_0.pdf

[5] Kaanto R, Kagira J, Waititu K, Ngotho M, Maina N, Gachohi J. Characteristics of patients presenting with septic wounds in selected hospitals in Kajiado County, Kenya. Int J Community Med Public Health. 2021;8(8):3743–9.

[6] Pieper B, Sieggreen M, Nordstrom CK, Freeland B, Kulwicki P, Frattaroli M. Discharge knowledge and concerns of patients going home with a wound. J Wound Ostomy Continence Nurs. 2007;34(3):245–53. 17505242 10.1097/01.WON.0000270817.06942.00

[7] Dawer A, Msengi V, Mtui TB, Sarakikya S, Suleiman R, Lusingu JPA. Antimicrobial resistance of bacterial isolates among patients with chronic wound infections in Tanga Regional Referral Hospital, Tanzania. Unpublished manuscript. 2025.

[8] Yusuf HM, Daninga PD, Li X. Determinants of rural poverty in Tanzania: Evidence from Mkinga District, Tanga Region. Deve Country Stud. 2015;5(6):40–8.

[9] Ishengoma DRS, Rwegoshora RT, Mdira KY, Kamugisha ML, Anga EO, Bygbjerg IC, et al. Health laboratories in the Tanga region of Tanzania: the quality of diagnostic services for malaria and other communicable diseases. Ann Trop Med Parasitol. 2009;103(5):441–53. doi: 10.1179/136485909X451726 19583914

[10] Global Data Lab. Health indicators, version v1.0. Nijmegen (NL): Global Data Lab, Institute for Management Research, Radboud University; Available from: https://globaldatalab.org/health/

[11] Gould L, Abadir P, Brem H, Carter M, Conner-Kerr T, Davidson J, et al. Chronic wound repair and healing in older adults: current status and future research. Wound Repair Regen. 2015;23(1):1–13. doi: 10.1111/wrr.12245 25486905 PMC4414710

[12] Alhassan AR, Kuugbee ED, Der EM. Surgical Healthcare Workers Knowledge and Attitude on Infection Prevention and Control: A Case of Tamale Teaching Hospital, Ghana. Can J Infect Dis Med Microbiol. 2021;2021:6619768. doi: 10.1155/2021/6619768 33981370 PMC8088377

[13] Woo KY, Wong J, Rice K, Coelho S, Haratsidis E, Teague L, et al. Patients’ and clinicians’ experiences of wound care in Canada: a descriptive qualitative study. J Wound Care. 2017;26(Sup7):S4–13. doi: 10.12968/jowc.2017.26.Sup7.S4 28704169

[14] Dorai AA. Wound care with traditional, complementary and alternative medicine. Indian J Plast Surg. 2012;45(2):418–24. doi: 10.4103/0970-0358.101331 23162243 PMC3495394

[15] Asuzu CC, Akin-Odanye EO, Asuzu MC, Holland J. A socio-cultural study of traditional healers’ role in African health care. Infect Agents Cancer. 2019;14(1). 10.1186/s13027-019-0232-y PMC6585125 31249608

[16] Albertyn R, Berg A, Numanoglu A, Rode H. Traditional burn care in sub-Saharan Africa: a long history with wide acceptance. Burns. 2015;41(2):203–11. doi: 10.1016/j.burns.2014.06.005 25062977

[17] Tyavambiza C, Dube P, Goboza M, Meyer S, Madiehe AM, Meyer M. Wound Healing Activities and Potential of Selected African Medicinal Plants and Their Synthesized Biogenic Nanoparticles. Plants (Basel). 2021;10(12):2635. doi: 10.3390/plants10122635 34961106 PMC8706794

[18] Taber JM, Leyva B, Persoskie A. Why do people avoid medical care? A qualitative study using national data. J Gen Intern Med. 2015;30(3):290–7. doi: 10.1007/s11606-014-3089-1 25387439 PMC4351276

[19] Jan M, Almutairi KH, Aldugman MA, Althomali RN, Almujary FM, Abu Mughaedh NA, et al. Knowledge, attitudes, and practices regarding wound care among general population in Aseer region. J Family Med Prim Care. 2021;10(4):1731–6. doi: 10.4103/jfmpc.jfmpc_2331_20 34123920 PMC8144783

[20] Van Wyk B-E, Moteetee NA. Ethnobotanical research in sub-Saharan Africa – documenting and analysing indigenous knowledge about medicinal, edible and other useful plants. S Afr J Bot. 2019;122:1–2. doi: 10.1016/j.sajb.2019.04.020

[21] R A C P, M B S. Knowledge, Attitudes and Practices of Patients Regarding Chronic Wound Care and Point Prevalance of Chronic Wounds at Surgical and Medical Units in Teaching Hospital Karapitiya, Sri Lanka. IOSR J Pharm Biol Sci. 2017;12(02):38–46. doi: 10.9790/3008-1202023846

[22] Bejarano-Roncancio JJ, M E, Saurith-López V, Sussman-Peña OA. Rev Fac Med [Internet]. 2025;59:3–11. Available from: http://www.scielo.org.co/scielo.php?pid=S0120-00112011000500002&script=sci_abstract&tlng=pt

[23] Van Hecke A, Grypdonck M, Defloor T. A review of why patients with leg ulcers do not adhere to treatment. J Clin Nurs. 2009;18(3):337–49. doi: 10.1111/j.1365-2702.2008.02575.x 19191982

