# Supplementary material for: Patient knowledge, attitudes and practices on chronic wound infections in Tanga Regional Referral Hospital, Tanzania; A qualitative study
Source: PLOS Glob Public Health. 2026 Feb 24;6(2):e0004698. doi: 10.1371/journal.pgph.0004698 (PMC12931743; doi:10.1371/journal.pgph.0004698)
Supplement: S1 Text — (DOCX) [file pgph.0004698.s001.docx]

Appendix A: Voices of Experience: Thematic Categorization of Participant Testimonies on Health Challenges and Practices.

*All names or identifying labels used in participant quotes are pseudonyms, indicated by quotation marks, to protect participant confidentiality. Any direct references to specific locations have been replaced with [Redacted].*

| **Voices of Experience: Thematic Categorization of Participant Testimonies on Health Challenges & Practices** |
| --- |
| **Delay in treatment-seeking** |
| "I came late to the hospital, believing this was just a fungus…" (P1, Female) |
| "It took like six months without going to the hospital…" (P2, Male) |
| “Yes, I was late to go to the hospital, I think I should have been when I started scratching  myself …” (P4, Female) |
| “Sometimes you reach a point where you give up because when you apply it, you see no results…it deprives you of strength…” (P2, Male). |
| "If it were, say, identified early, and I had gone to the hospital sooner, I wouldn't have reached this stage. They (traditional healers) delayed me, yes…" (P2, Male) |
| “Unfortunately, it started as a fungus, so I thought it was a fungus. I treated with antifungal drugs because most of the time fungi appeared on the feet, at the end of the day, the leg became swollen and started to turn a little brown, then I knew there was a problem …” (P9, Male) |
| **Wound Management** |
| “When I go to shower, because it started here at the bottom, I clean it, and then I apply medicine, thinking it's a fungus. It was negligence, thinking it's just a fungus because I didn't trip, wasn't pricked by a thorn, or cut myself…When I noticed... uh... my foot was swelling, that's when my children, who don't live with me, got alarmed and took me to the hospital” (P1, Female). |
| “I used to go to the pharmacy and buy a sterile gauze, I clean it and then I apply the medicine knowing that it is just a fungus… I was covering my wound with a piece of cloth like this so as to protect myself from the sand and step on… believing that it won’t affect anything …[I used] home clothes, my child” (P1, Female). |
| “I pour iodine on the gauze and then clean the wound. After cleaning, I apply the same iodine. Just once a day, mainly when I take a shower” (P1, Female). |
| Ah, for the first month I cleaned it every day, but in the following month, I would skip a day and then go dress it, like dress it today, skip tomorrow, then go the day after. I was unable to go frequently because of the financial challenge…I had to pay five thousand for dressing and four thousand for transport costs to reach the health centre every day … (P4, Female). |
| “…The lack of a cure for diabetes is a big problem, it’s humiliating, it means you get a toe amputated today, and a leg tomorrow” (P5, male). |
| “You never cleaned the wound yourself?” - (PM).  “Uh uh (disapproving)” (P3, Male). |
| “They [redacted hospital] cleaned me well, gave me medicine, a lot of blood transfusion, it started forming flesh properly, and finally I was discharged [after four months], the skin had attached well, I stayed at home, but because I'm from Kigoma, I went back home and after about four months, the condition started forming pus again, inside the leg, and the situation reappeared again. And it reappeared again, and that's the condition I'm in now” (P2, Male). |
| “I cleaned [the wound] myself. I just cleaned it using water or sometimes I cleaned it using the same healer's medicines, like if someone gives you water to mix, so I cleaned it then later there are other medicines he gives you to apply… once a day” (P2, Male). |
| “I was just washing it with metronidazole and dressing it with honey” (P4, Female). |
| “I continued using these ordinary medicines like paracetamol” (P10, Male). |
| **Ritual Practices** |
| “I visited like eight traditional healers…after using traditional medicine for three years…the condition became worse…you can apply medicine today, until tomorrow the leg will be sore and itchy…” (P2, Male). |
| “The second (traditional healer) said something like... I don't know... there are…two birds with two beaks, that's why you have sores here and there, that's what's troubling you. (Laughter)…the third traditional healer [said] it's because of spirits... I don't know, they want me to become a traditional healer, so I need to perform rituals to become a healer and that condition will go away..each one came with their own reason. He claimed...(long pause)... that I had stepped on, I don't know, mermaid children on the road... at a crossroads…” (P2, Male). |
| “I used the bark of a tree called Mzambarao… I would feel less tired after drinking it, and my frequent urination reduced. [I drank it] twice a day, two small cups…I got that advice from another diabetic patient…effectiveness was…equivalent to the pills because it lowered the sugar level faster…The pills did lower it, I don't deny, but their action was a bit slower” (P6, male). |
| “These Western medicines from the hospital didn't work. Every time I take these hospital medicines I don’t get relief, I met a gentleman, he said let me give you medicine. He gave me a leaf and I chewed it and put it on my wound” (P9, Male). |
| “A belief in what? Superstition. That is, they don't believe that the problem was caused by God but immediately see it as caused by another human. So they can't solve it without resorting to superstition” (P1, Female). |
| “We used to drink herbal medicines a long time ago but not for wounds. Since I got burned, I've never taken herbal medicines” (P3, Male). |
| “Yeah, some healers tell you to use [the medicine] three times a day if it's for drinking, and for ointments, there are some that you apply to the wound, you apply it. He might tell you to apply it today, then clean it tomorrow and reapply, or apply in the afternoon and then in the evening. The medicines are so strong, they itch and hurt all day” (P2, Male). |
| “Diabetes started for me in 2011…unfortunately, we have this problem in my family. My younger brother told me, "Forget the hospital medications. Look for herbal remedies." I started using those Chinese herbs, until 2016 I couldn't find them anymore. I completely missed them. Then, fortunately, although I had used many medicines before and saw they didn't work. Many turned out to be lies, someone tells you three hundred thousand TSh, then you leave them. I used many. But around 2016, I met an old man in the village. That man has good medicine, really. For diabetes, eh, I appreciate it. He gave me these large capsules for a month, I could take them and stay well for a year. Without needing anything else” (P9, Male). |
| “He gives you roots. There are many types of roots. So he cuts them and gives you up to seven days' worth. So you take it for a month or two. It's a very good medicine. I really appreciate that it's the medicine for diabetes, yes” (P9, Male). |
| **Eating Behavior** |
| “At night... we eat light foods like bananas… or cassava with tea” (P1, Female). |
| “I had never followed any diet, I eat ugali, rice and sometimes I miss the food completely…I eat meat but once in a while depending on the economic situation, if I get it today I might go a whole month without it again” (P2, Male). |
| “I often don't eat at lunch” (P4, Female). |
| “In the morning, I can only drink porridge...And eat nothing until the next morning...I can’t taste food...But surprisingly, the sugar level doesn't stabilize” (P5, Male). |
| “In the morning sometimes I might have tea, sometimes I might miss it, yes if I get it, in the afternoon I eat ugali, if I get rice I eat, I don't have a specific diet, maybe this diet is because I haven't got it for my health yet” (P2, Male). |
| “I usually eat boiled bananas...around 9 a.m and before I go to work, I must have a cup of this (indicating something) porridge. In the afternoon, I eat ugali. Sometimes at night, I eat potatoes, those kinds of boiled potatoes, yes” (P6, Male). |
| “First thing in the morning I drink millet porridge. In the afternoon, I cook dried bananas” (P10, Male). |
| **Inadequate Healthcare Services** |
| “No, [HCW’s did not give instructions on cleaning the wound] because they cleaned it themselves” (P1, Female). |
| “When I got to the hospital, they said let's rest her because she is diabetic, let's give her first aid, so I stayed from Saturday, Sunday, around Monday, Tuesday, then they said no, let's take her to the city, to the regional hospital for an X-ray, to understand the problem and how it's progressing” (P1, Female). |
| “Maybe just the biggest challenge is the delay in treatment. Since I arrived here on Tuesday evening, they have already set their treatment time table only for Tuesday and Wednesday of next week. So it bothered me and it was difficult to get treatment and I see that the bacteria are attacking more and more now…” (P9, Male). |
| “I went to the district hospital, I didn’t get any treatment there. I stayed there for four days, so I had to come back to the referral hospital …” (P10, Male). |
| “[The HCWs] tell us to eat good food, soup, small fish, spinach and vegetables” (P3, Male). |
| “At that time I just thought it was something ordinary that would heal even with local remedies. But after trying local treatments and failing, I started going to the health center. So even after reaching the health center, they would clean it, give me pills, injections, but it kept growing, worsening, and spreading over my leg” (P2, Male). |
| “I started feeling unwell about three months ago. It began as a blister, like I started itching, and then the blister burst and turned into a wound. The wound grew bigger and bigger, so I went to a private hospital. There, I was dressed and given medication, I would go home, get dressed again, and medicate, but... it seemed fine for a while, then it developed slough, so I had to come here” (P4, Female). |
| “[At the private hospital] I was using povidone, I was using... there are these drips, metronidazole (inaudible)... these small drips called metronidazole, for cleaning. It improved again but then deteriorated, like it developed some sort of fungus. [At the hospital] they did not give me any dietary instructions, only that after being dressed, when bathing, I should keep it dry. I wear a wrapper and elevate my leg while bathing to keep it dry. After a month I went to the district [redacted] hospital, but…the wound was not healing well, that's why I came to this [regional] hospital (TRRH)” (P4, Female). |
| “At the hospital, they dressed it with NS, I was cleaned with NS and then dressed with honey. Just once a day” (P4, Female). |
| “There’s a doctor there named Niko, we went... he was called to my home, he came and saw it and said it’s not a big deal, let’s just inject some drying injections. So, he injected me with five injections, but when they were finished, I still felt pain and the swelling increased. I [went to my] diabetes clinic, when I got there, they looked at it and said the foot might have been damaged so they gave me a transfer to go to Bombo (TRRH) and they removed my toe” (P5, Male). |
| “After being discharged on the 29th, I was given instructions on using... (long pause). I was told that I must use oil on the dressed foot...After one week, I was told to dress it using honey, and I did that. It was a daily clinic at [redacted]” (P5, Male). |
| “They gave me a medicine called povidone iodine. They gave me pieces of gauze and bandages. They told me to clean it first with spirit, and after cleaning it with spirit, then dress it with povidone. [I did this] twice a day, in the morning, but I didn't use spirit, I used warm water with salt” (P6, Male). |
| “The most important thing they [hospital] told me was to use honey. I have never used honey as a treatment, you see, it was something I didn't believe in. They did not give me any dietary guidelines” (P6, Male). |
| “Yes, I went to the health center, they told me to come every day to get dressed. They were applying that povidone, iodine. Yeah, I was applying in two places. Every day I applied povidone. But unfortunately, the wound started smelling worse. I applied povidone, and it smelled worse. But I also applied that leaf. The leaf refused to stay. That's the problem I had. Because there on the toe, I don't know if it's due to friction or what, the leaf just refused to stay. Every time I applied it, it fell off” (Patient 15) |
| **Living and coping with chronic wounds** |
| “Ah, challenges, truly... Ah, there aren't any, just worries. Anxiety because you know, often with diabetic wounds, you hear about people having their legs amputated…you just feel the pain, it's like pricking - Ah - it's sharp. Yes, but when you step on it - Ah - no problem, and secondly, the fluid it releases has a bad smell, so when you sit with others... you find flies following you, you can't sit comfortably” (P6, Male). |
| “The challenge was that my foot was swollen... it was badly swollen, so walking and wearing shoes or sandals was difficult” (P1, Female). |
| “I had a severe headache and I collapsed unconsciously. One leg was left outside, and the other was near the fire. So for about two hours, that leg was next to the fire. When I woke up, I was surprised to find one leg completely numb. When I touched it, my arm, neck, and side up to my neck were numb. They took me to [redacted] Hospital, our district hospital. When I got there, they tested me and said my blood pressure was high. They gave me medicine, and all the numbness subsided…When they saw the burn was severe, they referred me to Tanga Bombo. [Redacted hospital] saw they couldn't handle it because it's a small hospital” (P3, Male). |
| **“T**hen I came here, they took me and removed the dry flesh, I went to the theater, and they fixed an exposed bone. Yes, I've been treated, and now it's the wound that's bothering me. The main challenge with the wound is just the pain... it hurts, especially when being cleaned, until you take painkillers or get an injection, then it calms down” (P3, Male). |
| “I don't have insurance. My situation is humble. My children contribute, and we pay” (P3, Male). |
| “I'm stuck, yes, like the other day I went to the theater, I went to get grafts, they refused to take. Yes, that's why they came to tell me to take the fluids for research on the wound, maybe that is why it refused. Not that I'm refusing because I don't have a specific job” (P3, Male). |
| “Sometimes when you apply some medicines, like if you apply it today, you can stay with it almost from today to tomorrow or the day after, and that leg still hurts and itches, it pulls and makes you uncomfortable, so challenges like that, I had no peace at all” (P2, Male). |
| “The challenge with the schedule was sometimes he might tell you to do this or he comes and you do this, sometimes you don't follow exactly what he suggests [because] sometimes you reach a point where you give up because when you apply it, you see no results, that's why you think maybe if someone else appears because maybe I should leave this one first, because there's that challenge, it deprives you of strength, even the appetite to eat sometimes, you don't eat because of the suffering you get from the pain of those medicines” (P2, Male). |
| “I've been given instructions, but the (stuttering) economic situation is challenging. I think it [treatment] can even reach three hundred thousand. Because they told me the first one, they said I don't know one hundred and fifty thousand... one hundred I don't know and... One hundred, I don't know and bit.. Something like that, then the second one for scraping they said one hundred and fifty thousand. The plan here, I think maybe after I finish being given blood because I... I came in here because I was lacking blood, so I think maybe after I finish the blood transfusion, my schedule as advised, maybe I leave first maybe to look for... people to organize, if money is found then I come and get this issue handled” (P2, Male). |
| “I really believe if I had gone to the hospital earlier I wouldn't be in this condition…I just believed it would heal in time, but it wasn't as I thought…it's not good to self-treat at home, because there are many things you don't understand. It's better to go to the hospital early to know the proper procedure-...the dangers are like this, the wound becomes big and you stay with it for a long time” (P4, Female). |
| “My body becomes very heavy, I can't handle it, yes. I just can't. For example, I might go outside, but it's a challenge. I might fall…It feels like my legs are weakening, yes. And, you might use the toilet but then find it hard to get up. In short, I can't manage on my own ” (P5, Male). |
| “My opinion is that more effort should be made to find a cure for diabetes…Yes, the lack of a cure for diabetes is a big problem, it’s humiliating, it means you get a toe amputated today, a leg tomorrow…it will be a continuous process now. Removing these body parts. If this foot is problematic, are there no other treatments other than amputation?” (P5, Male). |
| “I told them, look, this is not enough, I need to move forward, because now it's starting to produce fluid with a strong smell. I saw no progress staying there, why should I stay? What I did was just ask for a paper. Just give me a paper to go to Bombo. I feel like we've failed here” (P6, Male). |
| “I sometimes take a crepe bandage, it's light, but I just wrap it lightly around, so I can sit with others inside there and talk to them without flies bothering me” (P6, Male). |
| “If there was a treatment, people wouldn't have limbs amputated, there wouldn't be removal of body parts, but now it has become a critical issue. Yes, sir. An issue that is now being talked about a lot, eh. And also, people have continued to be... You know, a disease is not rejected, it's accepted because it has already reached you, but now people are scared of it, eh, they are afraid. So, I continue to pray that you continue researching so we can find medicines that can cure these wounds” (P6, Male). |
| “These sugar testing screens, they are expensive. But without testing you can never know. And when you come to the hospital you are told there are no sticks, no this or that. So it's better to have your own” (P9, Male). |
| “Ah, I don’t really understand the medications I take” (P10, Male). |
| **Suggestions from patients** |
| “My opinion to my fellow Tanzanians is that when we get a minor wound, we shouldn't ignore it but rush to the hospital” (P1, Female). |
| "Ah, my suggestion, which I had, first is to thank them, to thank them for continuing to try to research, yes, in order to help more diabetic patients like us, especially when we get wounds like these, if possible to find a treatment" (P6, Male). |
| “My opinion is that more effort should be made to find a cure for diabetes…the lack of a cure for diabetes is a big problem, it’s humiliating, it means you get a toe amputated today, a leg tomorrow…it will be a continuous process now. Removing these body parts…people aren't delving deeply enough technically because if this foot is problematic, are there no other treatments other than amputation? That's my question” (P5, Male). |
| “Uh, other recommendations, it's not good to self-treat at home, because there are many things you don't understand. It's better to go to the hospital early to know the proper procedure- the dangers are like this, the wound becomes big and you stay with it for a long time” (P4, Female). |
| “Ah, we thank you for your attention. You've come to listen to us, I believe even this is a treatment for us... we learn...I know even when you get your research I'll have been helped. Suggestions maybe just continue helping us, once you get your research come help us..help us so we can go back to the streets… I just pray God helps you continue being this way” (P2, Male). |
