# Supplementary material for: Patient knowledge, attitudes and practices on chronic wound infections in Tanga Regional Referral Hospital, Tanzania; A qualitative study
Source: PLOS Glob Public Health. 2026 Feb 24;6(2):e0004698. doi: 10.1371/journal.pgph.0004698 (PMC12931743; doi:10.1371/journal.pgph.0004698)
Supplement: S2 Text — (DOCX) [file pgph.0004698.s002.docx]

**Semi-Structured Survey on Wound Healing Practices**

**Section 1: Demographic Information**

1. Name: ___________________
2. Date of interview:____________________
3. Date of Birth (DOB): ___________________
4. Age in years (if DOB not known): ______________________
5. Gender: ⃝ Male ⃝ Female
6. Occupation: _______________________
7. Religion: __________________________
8. Type of wound (select which is applicable):

⃝ Diabetic Foot Ulcer

⃝ Surgical-Site Infection

⃝ Cellulitis

⃝ Septic wound

⃝ Malignant Ulcer

⃝ Fungal Infection

⃝ Other ______________

Wound Duration:

**Section 2: Interview Guide**

**Dietary Habits**

1. Can you please explain your regular diet? **Probe:**
2. Do you follow any special diet believing it might help in healing your wound faster?

If yes/no, please describe:

**Adherence to Medication**

1. How regularly do you take the medications prescribed to you? **Probe:**

⃝ Always

⃝ Most of the time

⃝ Sometimes

⃝ Rarely

1. Have you faced any challenges in sticking to your medication schedule?

If yes/no, please describe:

**Use of Traditional Medicine/Herbal Remedies:**

1. Do you use any herbal remedies for wound care instead of, or alongside, your prescribed medication? **Probe:**

If yes/no, please describe:

1. What are your beliefs or perceptions about the effectiveness of traditional medicine/herbal remedies in wound care? **Probe:**

**Wound Care Practices**

1. How often, if any, do you redress your wound? **Probe:**

⃝ Daily

⃝ Every other day

⃝ Weekly

⃝ Never

⃝ Other (please specify): __________________________________

1. Have you encountered any barriers to maintaining proper wound care? **Probe:**

If yes/no, please describe:

1. Do you have any comments to tell us, opinions or suggestions? Thank you.
