# Supplementary material for: Patient knowledge, attitudes and practices on chronic wound infections in Tanga Regional Referral Hospital, Tanzania; A qualitative study
Source: PLOS Glob Public Health. 2026 Feb 24;6(2):e0004698. doi: 10.1371/journal.pgph.0004698 (PMC12931743; doi:10.1371/journal.pgph.0004698)
Supplement: S1 Checklist — (DOCX) [file pgph.0004698.s003.docx]

COREQ (COnsolidated criteria for REporting Qualitative research) Checklist

| **Item** | **Response** | **Reported on Page/Section** |
| --- | --- | --- |
| **Domain 1: Research team and reflexivity** | | |
| **1. Interviewer/facilitator** | Bilingual co-author (P.M.) conducted all interviews. | Methods, Data Collection |
| **2. Credentials** | P.M. holds a Master’s degree and is trained in qualitative methods. She is a qualitative researcher at National Institute for Medical Research (NIMR) | Methods |
| **3. Occupation** | Qualitative researcher with the National Institute for Medical Research. Over 8 years of experience. | Methods |
| **4. Gender** | One female interviewer | Methods |
| **5. Experience and training** | Received training in qualitative interviewing and ethical research practices. | Methods |
| **6. Relationship established** | No prior relationship with participants before recruitment | Methods, Sampling Strategy |
| **7. Participant knowledge of interviewer** | Participants were informed of the study's purpose and that the interviewer was affiliated with the research team | Methods |
| **8. Interviewer characteristics** | Bilingual, culturally fluent, trained to reduce social desirability bias | Methods |
| **Domain 2: Study design** | | |
| **9. Methodological orientation and theory** | Phenomenological qualitative design. | Methods, Study Design |
| **10. Sampling** | Consecutive sampling with maximum purposive variation | Methods, Sampling Strategy |
| **11. Method of approach** | Face-to-face recruitment in hospital wards | Methods, Sampling Strategy |
| **12. Sample size** | 15 participants | Results, Sample Characteristics |
| **13. Non-participation** | No eligible participants declined | Methods, Sampling Strategy |
| **14. Setting of data collection** | Private rooms within Tanga Regional Referral Hospital | Methods, Data Collection |
| **15. Presence of non-participants** | No one else was present during interviews | Methods, Data Collection |
| **16. Description of sample** | Age, gender, religion, education, occupation, wound type/duration | Results, Table 1 |
| **17. Interview guide** | Semi-structured, open-ended guide piloted and refined after two pilot interviews | Methods, Interview Procedures |
| **18. Repeat interviews** | No repeat interviews conducted. | Methods, Interview Procedures |
| **19. Audio/visual recording** | Audio recording used. Participants were aware before signing consent form. | Methods, Data Collection |
| **20. Field notes** | Field notes recorded in NVivo after the interviews were conducted. | Methods, Data Collection |
| **21. Duration** | 25–35 minutes. | Methods, Interview Procedures |
| **22. Data saturation** | Saturation discussed and confirmed | Methods, Data Analysis |
| **23. Transcripts returned** | Transcripts were not returned to participants due to logistical constraints | Methods, Limitations |
| **Domain 3: analysis and findings** | | |
| **24. Number of data coders** | Three coders (P.M., V.M., A.D.) | Methods, Data Analysis |
| **25. Description of the coding tree** | Themes and subthemes described and structured in Table 2 | Results, Table 2 |
| **26. Derivation of themes** | Both deductive and inductive themes | Methods, Data Analysis |
| **27. Software** | NVivo | Methods, Data Analysis |
| **28. Participant checking** | No participant feedback on findings was collected | Methods, Limitations |
| **29. Quotations presented** | Yes, with participant identifiers (e.g., P2, P6). | Results, Thematic Sections |
| **30. Data and findings consistent** | Yes, themes supported with quotations | Results |
| **31. Clarity of major themes** | Clearly presented in Results and Table 2 | Results, Table 2 |
| **32. Clarity of minor themes** | Discussion includes minor variations across participant groups | Results, Thematic Interpretation |
